# Supplementary material for: ATP modulates self-perpetuating conformational conversion generating structurally distinct yeast prion amyloids that limit autocatalytic amplification
Source: J Biol Chem. 2023 Mar 28;299(5):104654. doi: 10.1016/j.jbc.2023.104654 (PMC10149227; doi:10.1016/j.jbc.2023.104654)
Supplement: Supporting Figures S1–S4 [file mmc1.pdf]

Supporting Information

ATP modulates self-perpetuating conformational conversion generating structurally distinct yeast prion amyloids that limit autocatalytic amplification

Sayanta Mahapatra,[1,2,#] * Anusha Sarbahi,[1,2,#] Neha Punia,[1,2,¶] Ashish Joshi,[1,2] Anamika Avni,[1,3] Anuja Walimbe,[1,2] and Samrat Mukhopadhyay[1,2,3] *

[1]Centre for Protein Science, Design and Engineering, [2]Department of Biological Sciences, and [3]Department of Chemical Sciences, Indian Institute of Science Education and Research (IISER) Mohali, Punjab, India

[#]Contributed equally

*Corresponding authors: sayanta12@gmail.com and mukhopadhyay@iisermohali.ac.in

[¶]Present address: The City University of New York, NY, USA

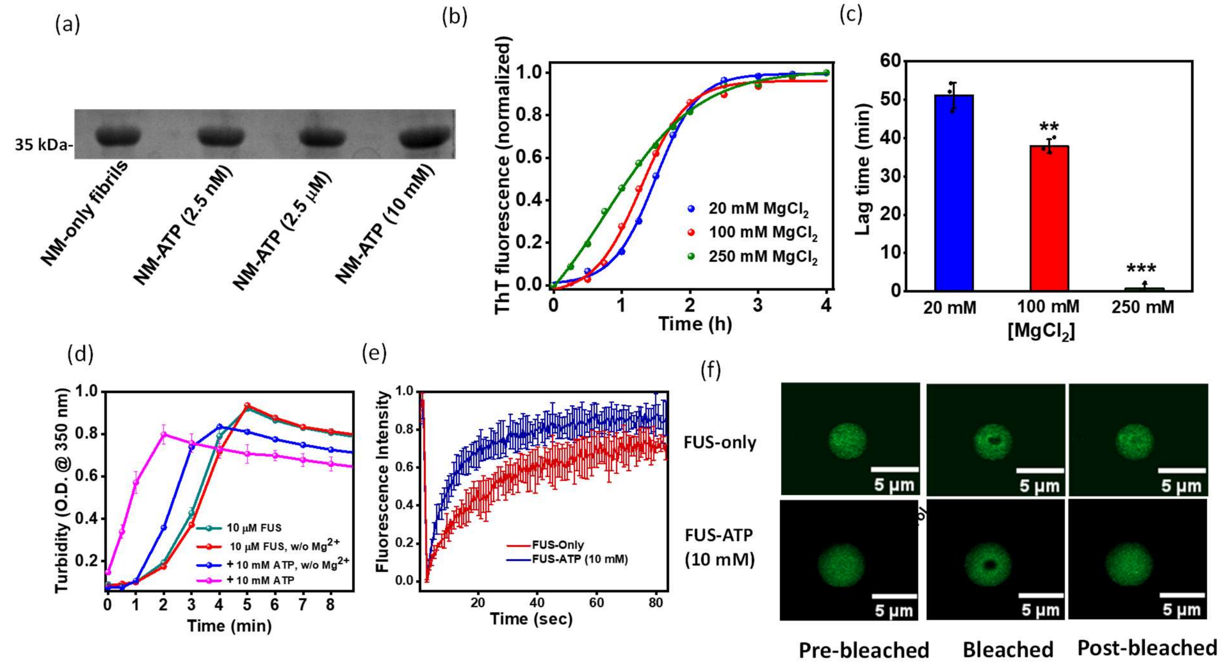

Figure S1. (a) NM or NM-ATP fibrils sedimented at 16,400 rpm for 30 minutes and pellets were resuspended in 8 M urea (20 mM tris, pH 7.4) and incubated overnight prior to SDS-PAGE analysis. (b) Representative normalized ThT fluorescence kinetics of rotated (80 rpm) NM (2.5 μM) aggregation in the presence of 20 mM, 100 mM, and 250 mM $MgCl_2$ at room temperature. (c) Lag times of rotated (80 rpm) aggregation of NM monomers (2.5 μM) in the presence of 20 mM, 100 mM, and 250 mM $MgCl_2$ at room temperature. Standard deviations were calculated from three independent replicates (n = 3), $P < 0.01$, $P < 0.001$ for lag times in presence of 100 mM and 250 mM $MgCl_2$, respectively, compared to the lag time in the presence of 20 mM $MgCl_2$. (d) Liquid-liquid phase separation (LLPS) of FUS (10 μM) in the absence of ATP. LLPS of FUS (10 μM) without $MgCl_2$.Also, the liquid-liquid phase separation of FUS (10 μM) in the presence of 10 mM ATP in the absence or presence of $MgCl_2$ was monitored by the turbidity assay (at 350 nm). Standard deviations were calculated from three replicates (n = 3). (e) FRAP kinetics of FUS (10 μM) droplets formed in the absence or presence of 10 mM ATP. The solid lines represent the fitted curves. (f) The fluorescence images of FUS or FUS-ATP (10 mM) droplets during FRAP measurements are shown.

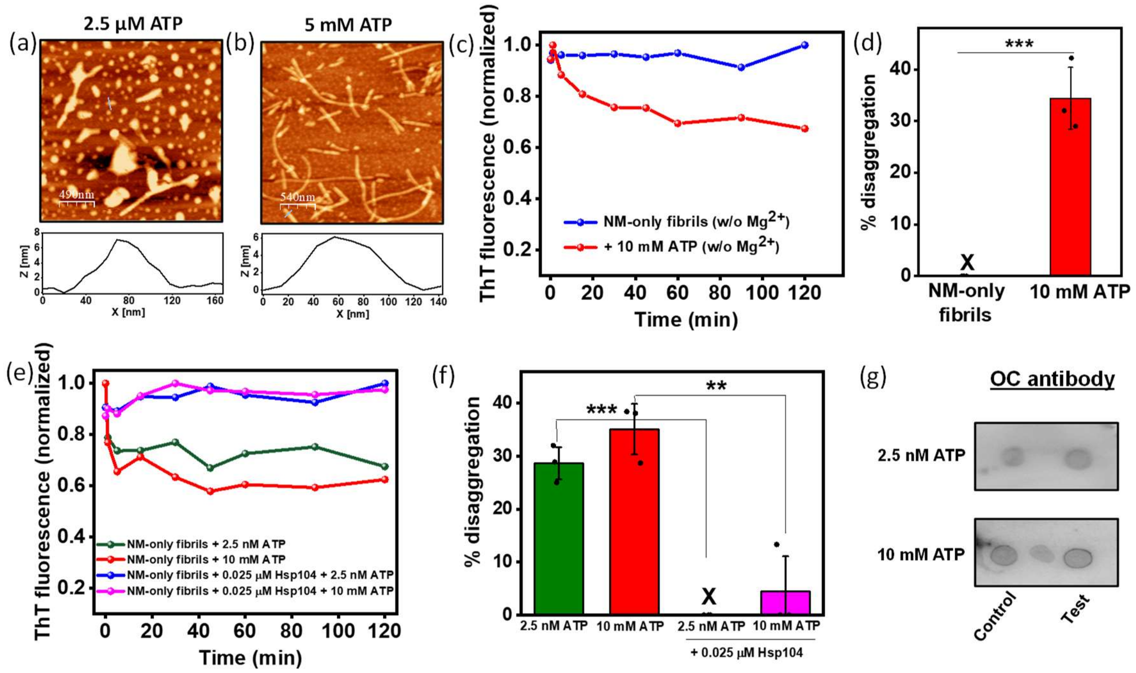

Figure S2. (a,b) AFM images showing disaggregation of amyloids formed from rotated (80 rpm) aggregation reaction of NM (2.5 μM) at room temperature by (a) 2.5 μM ATP and (b) 5 mM ATP with a height of ~ 6 nm. (c) Representative disaggregation kinetics of NM-only fibrils (2.5 μM monomers) without or with 10 mM ATP in the absence of $MgCl_2$ at room temperature and 80 rpm, showing the percentage of disaggregation in Figure S2d. Standard deviations were calculated from three independent replicates (n = 3), $P < 0.001$ for disaggregation by 10 mM ATP compared to NM-only fibrils. (e) Representative disaggregation kinetics of NM-only fibrils (2.5 μM monomers) by ATP in the absence or presence of Hsp104 (0.025 μM) at room temperature and 80 rpm. (f) Percentage of disaggregation of NM-only fibrils (2.5 μM monomer) formed at room temperature and 80 rpm by ATP in the absence or presence of Hsp104 (0.025 μM). Standard deviations were calculated from three independent replicates (n = 3), $P < 0.001$ for disaggregation by 2.5 nM ATP in the presence of Hsp104 compared to in the absence of Hsp104; $P < 0.01$ for disaggregation by 10 mM ATP in the presence of Hsp104 compared to in the absence of Hsp104. (g) NM-only fibrils (2.5 μM monomer) were spotted before and after disaggregation by 2.5 nM and 10 mM ATP on the nitrocellulose membrane and were probed using the OC antibody.

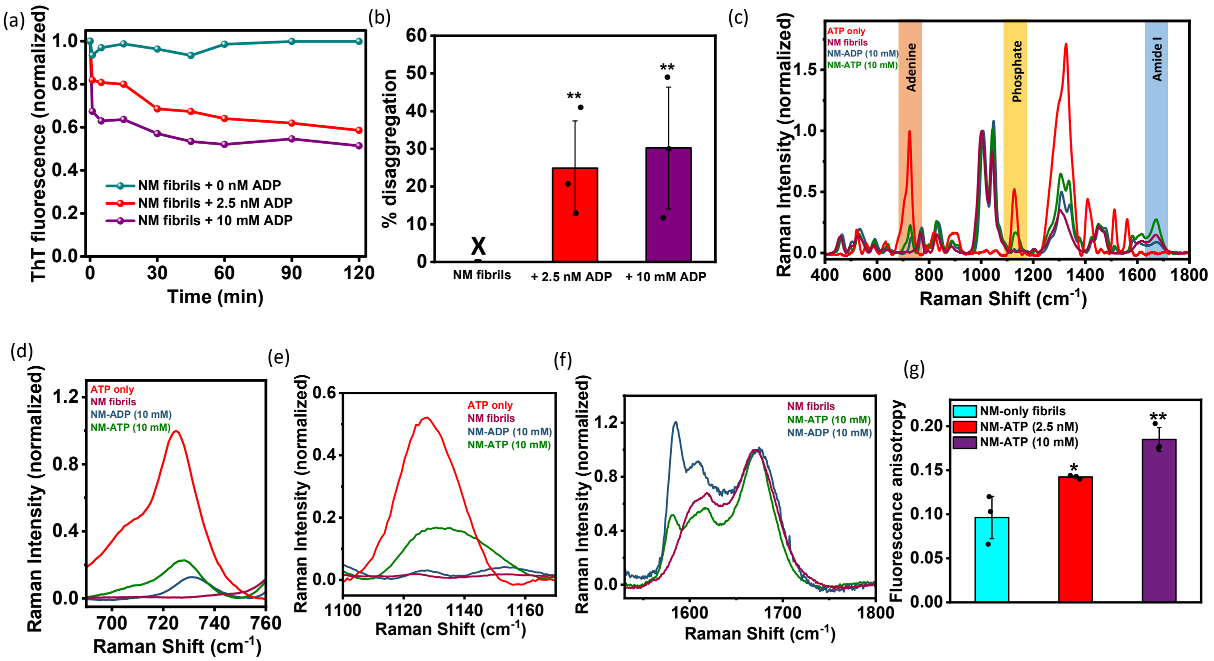

Figure S3. (a) Representative disaggregation kinetics of NM-only fibrils (2.5 μM monomers) without or with ADP at room temperature and 80 rpm. (b) The percentage of disaggregation. Standard deviations were estimated from three independent replicates ($P < 0.01$) for the percentage of disaggregation with respect to no ADP. (c) Raman spectra of NM-only fibrils (2.5 μM monomers) and NM-ADP fibrils formed in the presence of ADP with 2.5 μM NM monomers highlighting the adenine (brown), phosphate (yellow), and amide I (blue). The peaks of adenine and phosphate moieties are shown at (d) 725 cm^{-1} and (e) 1127 cm^{-1}, respectively, for free ADP or NM-ADP fibrils. (f) The amide I region of NM-only or NM-ADP fibrils, as highlighted in blue in Figure S3c. (g) Steady-state fluorescence anisotropy of fluorescein-5-maleimide-Cys150-NM amyloid fibrils formed in the absence or presence of different concentrations of ATP. Standard deviations were calculated from three independent replicates (n = 3), $P < 0.05$ in the presence of 2.5 nM and $P < 0.01$ in the presence of 10 mM ATP compared to NM-only fibrils.

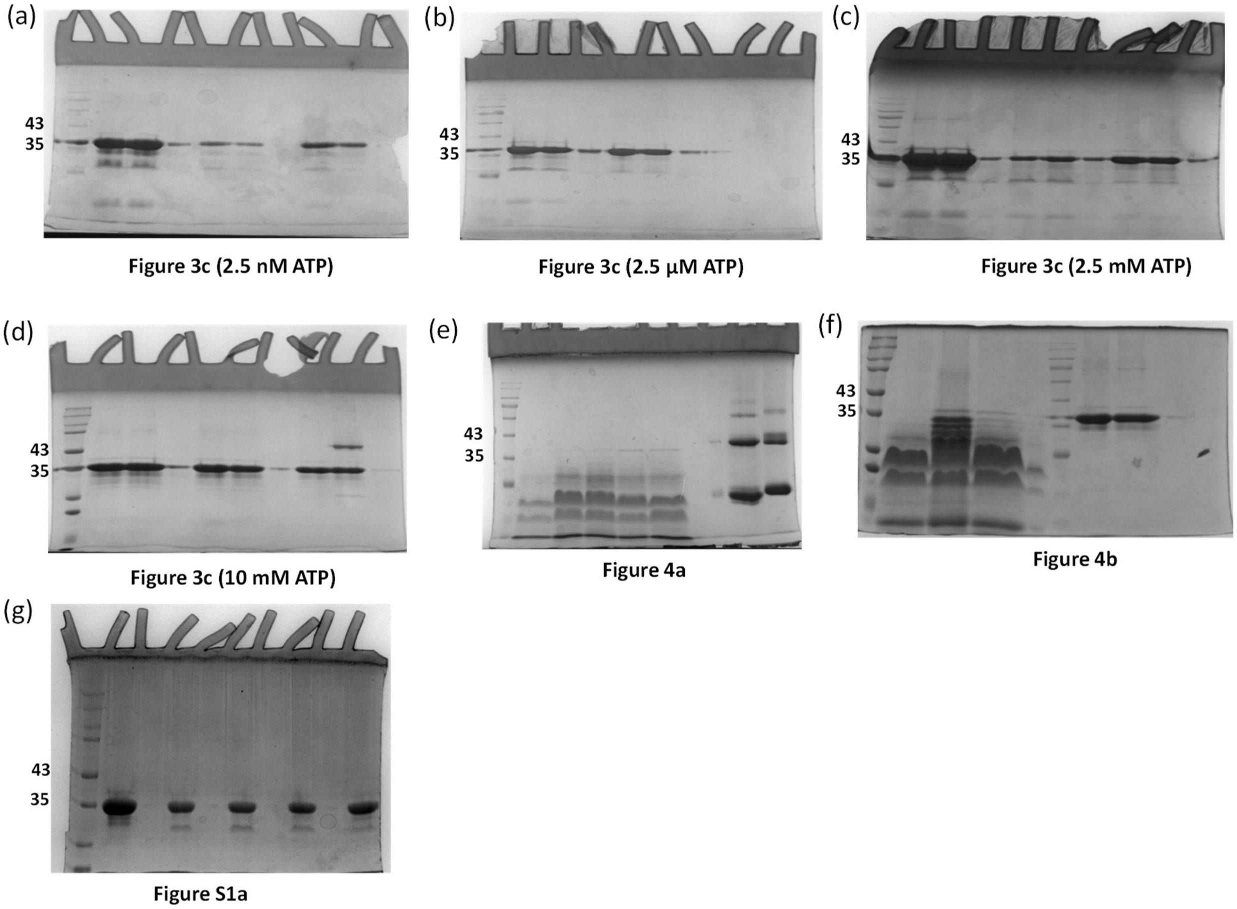

Figure S4. Full uncropped gels indicating the molecular weight marker (kDa) on the left.
